# Analysis of secondary structural elements in human microRNA hairpin precursors

**DOI:** 10.1186/s12859-016-0960-6

**Published:** 2016-03-01

**Authors:** Biao Liu, Jessica L. Childs-Disney, Brent M. Znosko, Dan Wang, Mohammad Fallahi, Steven M. Gallo, Matthew D. Disney

**Affiliations:** Department of Chemistry, Scripps Florida, The Scripps Research Institute, 130 Scripps Way 3A1, Jupiter, FL 33458 USA; Department of Chemistry, Saint Louis University, 3501 Laclede Ave, St. Louis, MO 63103 USA; Department of Biostatistics, Roswell Park Cancer Institute, 701 Ellicott St, Buffalo, NY 14203 USA; NYS Center of Excellence in Bioinformatics and Life Sciences, 701 Ellicott St, Buffalo, NY 14203 USA; Informatics Core, The Scripps Research Institute, 130 Scripps Way 2B2, Jupiter, FL 33458 USA; The State University of New York at Buffalo, Buffalo, NY 14203 USA; Center for Personalized Medicine, Roswell Park Cancer Institute, Buffalo, NY 14263 USA

## Abstract

**Background:**

MicroRNAs (miRNAs) regulate gene expression by targeting complementary mRNAs for destruction or translational repression. Aberrant expression of miRNAs has been associated with various diseases including cancer, thus making them interesting therapeutic targets. The composite of secondary structural elements that comprise miRNAs could aid the design of small molecules that modulate their function.

**Results:**

We analyzed the secondary structural elements, or motifs, present in all human miRNA hairpin precursors and compared them to highly expressed human RNAs with known structures and other RNAs from various organisms. Amongst human miRNAs, there are 3808 are unique motifs, many residing in processing sites. Further, we identified motifs in miRNAs that are not present in other highly expressed human RNAs, desirable targets for small molecules. MiRNA motifs were incorporated into a searchable database that is freely available.

We also analyzed the most frequently occurring bulges and internal loops for each RNA class and found that the smallest loops possible prevail. However, the distribution of loops and the preferred closing base pairs were unique to each class.

**Conclusions:**

Collectively, we have completed a broad survey of motifs found in human miRNA precursors, highly expressed human RNAs, and RNAs from other organisms. Interestingly, unique motifs were identified in human miRNA processing sites, binding to which could inhibit miRNA maturation and hence function.

**Electronic supplementary material:**

The online version of this article (doi:10.1186/s12859-016-0960-6) contains supplementary material, which is available to authorized users.

## Background

MicroRNAs (miRNAs) regulate gene expression via targeting mRNAs for destruction or translation repression [1–4]. Aberrant miRNA expression is associated with diseases [5, 6] including cancers [7], cardiovascular diseases [8], and HIV [9, 10]. In addition to being employed to explore mRNA and protein function *in vivo* [5, 11], miRNAs are also being explored as therapeutic targets [12, 13], in particular because overexpression of oncogenic miRNAs aids initiation and progression of various tumors [14–16]. Different strategies have been used to inhibit oncogenic miRNAs, including antisense or sponge oligonucleotides that bind mature miRNAs [17, 18] and inhibiting miRNA processing with small molecules [19–21]. A major liability of oligonucleotide-based therapeutics is poor tissue-specific delivery and cellular uptake [17]. Small molecules have been neglected for targeting RNA in general because it was speculated that RNA structural flexibility leads to lack of binding specificity. However, recent successful examples of using small molecules to target different RNAs [22, 23] have stimulated increasing interests in using small molecules to target miRNAs.

**Fig. 1:**
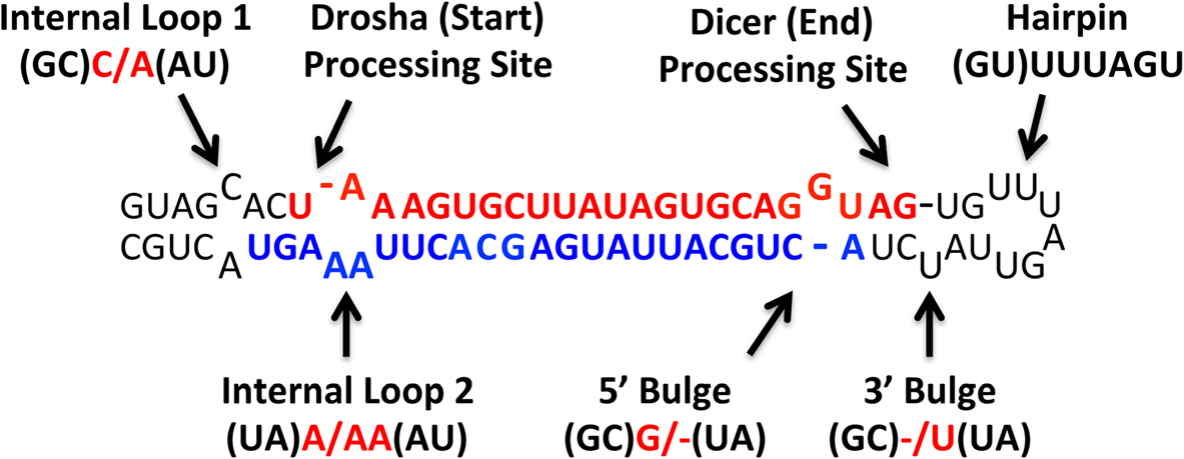
Schematic of the stem-loop structure of hsa-miR-20a. Red letters indicate the mature miRNA; blue letters indicate the mature miRNA*. Possible motifs in an RNA include internal loops, 5’ bulges, 3’ bulges, hairpins, and multibranch loops (not shown). The loops are named by the identity of unpaired nucleotides and base pairs (indicated by parentheses). The two sides of a bulge or an internal loop are indicated with a “/”

Usually, small molecules bind to non-canonically paired regions of RNA [22], such as bulges, internal loops, and hairpin loops (Fig. 1), as they provide enlarged major grooves for small molecule entry and partially exposed bases that can be exploited to increase specificity [13, 24]. Thus, miRNA hairpin precursors, which fold into stem loop structures that display various types of loops (Fig. 1) [25], are ideal candidates for small molecule binding. MiRNA processing occurs in both the nucleus (via Drosha) and the cytoplasm (via Dicer/transactivating response RNA-binding protein (TRBP)) [26]. Therefore, small molecules that localize to either compartment could inhibit miRNA maturation.

The number of known miRNA sequences has expanded tremendously [27, 28] because of the development of deep-sequencing technology. To develop specific small molecules that inhibit the processing of a single or few miRNAs, it is essential to identify unique secondary structural elements, or motifs. That is, it is important to know which motifs occur and their frequencies. In this study, we built a database of motifs found in human miRNA secondary structures. We examined the frequency of these motifs and which motifs are preferred at processing sites. It is still a mystery how the Dicer/TRBP complex achieves accuracy in processing pre-miRNAs with such huge diversity (more than a thousand different sequences in human). MiRNA processing sites (where the miRNA strands are cleaved) are presumed to be important. This analysis was then completed for RNAs with known structures, including highly expressed human RNAs. We hope that this analysis will eventually help our understanding of miRNA processing and improve identification of potential target sites for small molecules.

## Methods

### MiRNA hairpin precursor sequences and structures

All *Homo sapiens* miRNA and mature miRNA sequences were obtained from miRBase v.17 [27] (http://www.mirbase.org/). The secondary structures of miRNA hairpin precursors were predicted by RNAstructure [29], which uses a free energy minimization algorithm [30]. Please note that miRNA hairpin precursor structure determination via free energy minimization is the standard in the field [25].

### Other RNA sequences and structures

A previously constructed database of other RNA structures was also analyzed in order to make comparisons to miRNAs [31]. The database contains 1349 RNAs including 123 small subunit rRNAs [32], 223 large subunit rRNAs [32, 33], 309 5S rRNAs [34], 484 tRNAs [35], 91 signal recognition particles [36], 16 RNase P RNAs [37], 100 group I introns [38, 39], and three group II introns [40]. We also analyzed highly expressed human RNAs with known structures including 5S rRNA, 16S rRNA, 23S rRNA, 7SL (signal recognition particle), RNase P RNA, U4/U6 snRNA, and 465 non-redundant tRNAs [41].

### Motif nomenclature

**Fig. 2:**
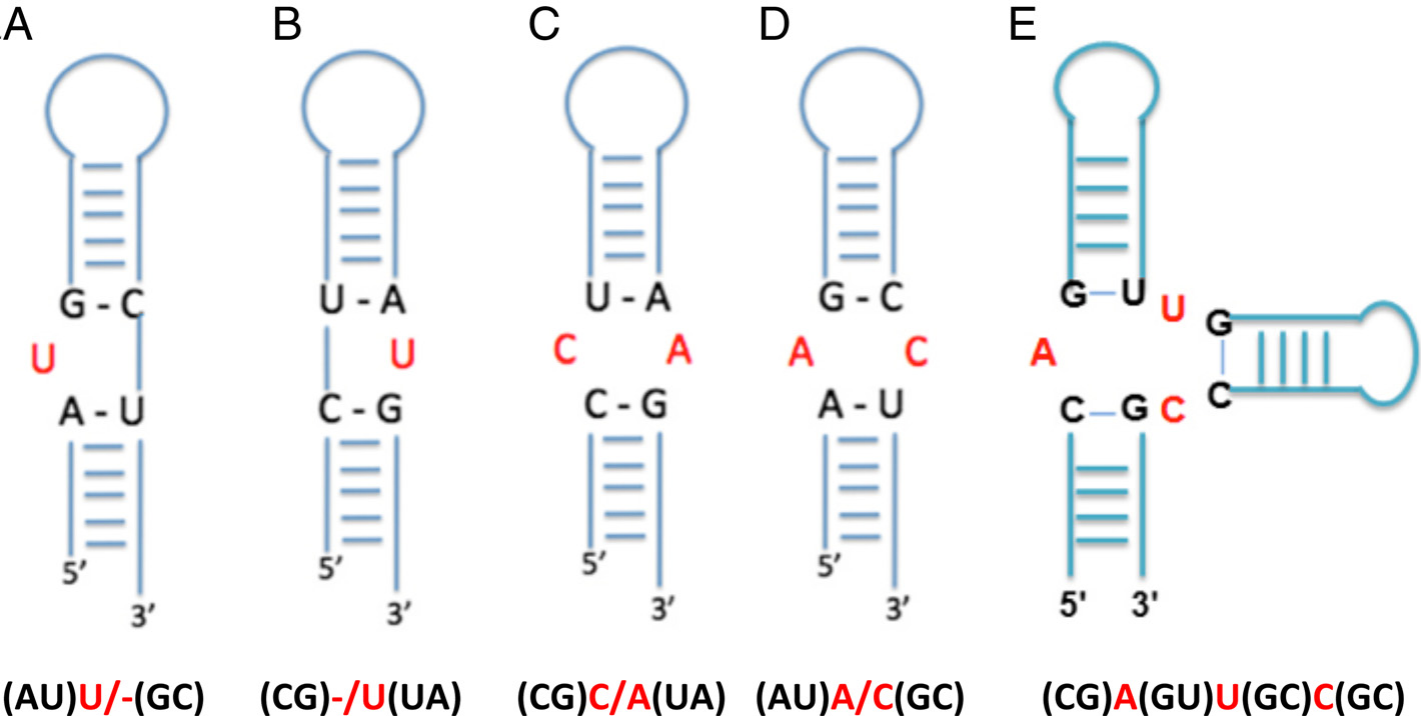
Diagrams of bulge and internal loops that have the same motifs but different orientations in miRNA hairpin precursor secondary structure (**a**–**d**), and a multibranch loop motif (**e**). (**a**) 5’ bulge loop (AU)U/-(GC), (**b**) 3’ bulge loop (CG)-/U(UA), (**c**) internal loop (CG)C/A(UA), (**d**) internal loop (AU)A/C(GC), and (**e**) multibranch loop (CG)A(GU)U(GC)C(GC). The motifs can be characterized by the identity of unpaired nucleotides (red letters) or the identity of unpaired nucleotides and closing base pairs (red and black letters). Note: (AU)U/-(GC) and (AU)-/U(GC) are different motifs. The equivalent 3’ bulge for the 5’ bulge (AU)U/- (GC) is (CG) -/U(UA)

The motifs predicted in miRNA hairpin precursor secondary structures include bulges, internal loops, hairpins, and multibranch loops (Figs. 1 and 2). Bulges are divided into two categories: 5’ bulge loops and 3’ bulge loops. Its designation as 5’ or 3’ is determined by the position of the unpaired nucleotide relative to the first hairpin loop in the miRNA's secondary structure (if it is 5’ to the hairpin loop or 3’).

A motif includes closing base pair(s) and non-canonically paired nucleotides. Sequences are always written 5’ – 3’. Closing base pairs are indicated with parentheses (for example, (GC)), and both nucleotides are always designated due to the possibility of GU pairs. The nucleotide 5’ to the loop is always listed first. Base pairs are listed at the beginning and end of the motif sequence for bulges and internal loops, only at the beginning of hairpin loops, and between all unpaired regions of multibranch loops. A “/” separates the two sides of bulges and internal loop. Please see Figs. 1 and 2 and the Results & Discussion for examples.

### Determination of statistical significance: are two motifs’ occurrence frequencies significantly different?

In order to determine if a particular motif is over- or under-represented, its statistical significance was calculated by a Z-score of type 1 error. That is, when Motif 1 occurred with probability p_1_ in a sample size n_1_, and Motif 2 occurred with probability p_2_ in a sample size n_2_, it is hypothesized that Motif 1 and Motif 2 occur with the same frequency. To reject this hypothesis, we calculate a Z-score using Eqs. 1 and 2:

1 $$ \phi =\frac{n_1\times {p}_1+{n}_2\times {p}_2}{n_1+{n}_2} $$

2 $$ Z- score=\frac{p_1-{p}_2}{\sqrt{\phi \left(1-\phi \right)\left(\frac{1}{n_1}+\frac{1}{n_2}\right)}} $$

If the Z-score >2, the hypothesis is rejected, and Motifs 1 and 2 have significantly different occurrence frequencies; if the Z-score <2, then no conclusion can be drawn.

### Determination of miRNA processing sites

The processing sites of a miRNA are defined as the first and last nucleotides in the mature miRNA. The mature miRNA was mapped onto miRNA hairpin precursors, and the motifs or paired regions containing the two end nucleotides were selected. If the site contains unpaired or non-canonically paired nucleotides, the processing site could be the unpaired nucleotides or the closing base pair. If the processing site is in a paired region, the base pairs next to the processing site are also included.

## Results and discussion

### A database of human miRNA hairpin precursor motifs

The number of human (*Homo sapiens*) miRNA sequences deposited in miRBase [27] has doubled in the past few years. As of August 2014, there were 1881 human miRNA sequences in miRBase. Although the secondary structures of most miRNAs have not been determined experimentally, a uniform system for miRNA annotation has been developed that employs secondary structure determination via free energy minimization [25, 29]. That is, the structures of miRNA hairpin precursors are accurately predicted from sequence. Therefore, RNAstructure [29], a free energy minimization algorithm that employs experimentally determined thermodynamic values, was used to predict the secondary structures of miRNA hairpin precursors. Only the lowest free energy structure was considered in our analysis. All non-canonically paired regions except the dangling ends for each hairpin precursor secondary structure were extracted and listed in the motif database. The database contains the following information for each motif: the miRNA ID/accession number, motif type (bulge, internal loop, hairpin, etc.), unpaired motif (single stranded nucleotides only), motif (unpaired nucleotides and the closing base pair(s)), and motif with closing base pairs and first non-nearest neighbor.

### Motif nomenclature

A motif includes unpaired or non-canonically paired regions (denoted in red) and its closing base pair(s) (denoted in black). Bulges and internal loops have two closing base pairs, hairpins have one closing pair, and multibranch loops have three or more. Examples of the nomenclature used are provided in Figs. 1 and 2. For example, the 5’ bulge loop in Fig. 1 is indicated as (GC)G/-(UA) while the 3’ bulge loop is named (GC)-/U(UA). Likewise, Internal Loop 1 is named (GC)C/A(AU); Internal Loop 2 is (UA)A/AA(AU); and the hairpin is named (GU)UUUAGU. For multibranch loops, the base pairs and the unpaired strands are written in order from 5’ to 3’ end. Since the 5’ closing base pair is also the 3’ closing base pair, it is repeated but in the opposite orientation. Thus, the multibranch loop in Fig. 2e is named (**CG**)A(GU)U(GC)C(**GC**) (5’ and 3’ closing base pairs denoted in bold). This nomenclature was developed such that the same unpaired regions with different closing base pairs can be distinguished from each other, for example (AU)U/-(GC) and (CG)-/U(UA); or (CG)C/A(UA) and (AU)A/C(GC) (Fig. 2).

### General survey of motifs in precursor miRNAs

**Fig. 3:**
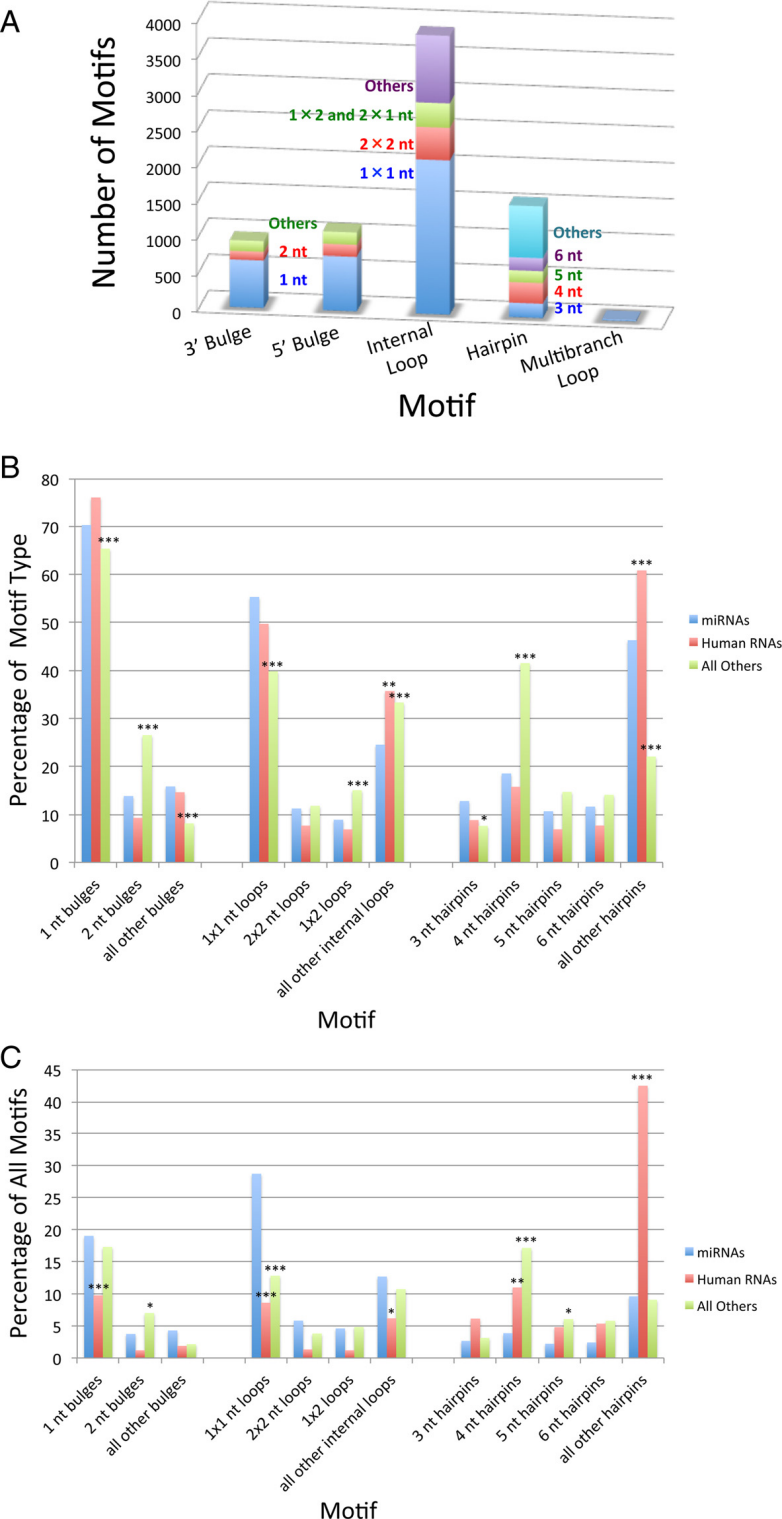
Comparison of motif types found in human miRNA precursors, highly expressed human RNAs, and RNAs with known structures from various organisms. (**a**) plot of the number of each secondary structural motif in human miRNA precursors including 3’ bulges (*n* = 924), 5’ bulges (*n* = 1089), internal loops (*n* = 3860), hairpins (*n* = 1546), and multibranch loops (*n* = 17). (**b**) plot of the percentage of each motif within its motif type (for example, the percentage of 1-nucleotide bulges of total bulges). (**c**) plot of the percentage of each motif. Total motifs: human miRNA precursor, *n* = 7436; highly expressed human RNAs, *n* = 2712; all other RNAs, *n* = 26,213; *, *p* <0.05; **, *p* <0.01; ***, *p* < 0.001

(A searchable database of motifs found in human miRNA hairpin precursors based on our analysis is available at: http://www.scripps.edu/disney/software.html.) The motifs present in miRNA hairpin precursor secondary structures are quite diverse. Of all miRNAs, only 32 (2.2 %) have fully paired stems (absence of non-canonically paired regions). The remaining 97.8 % have 1–14 motifs in the stem. There are a total of 7436 non-canonically paired motifs including 3862 internal loops, 1546 hairpin loops, 1089 5’ bulge loops, 922 3’ bulge loops, and 17 multibranch loops (Fig. 3a).

There are 2334 unique motifs (occur only once) if the base pairs and their orientations are not considered (31.4 % of total). If closing pairs and their orientations are considered, then there are 3808 unique motifs (51.2 % of total). Previous studies have shown that loop closing pairs can dramatically affect loop structure [42, 43]. Not surprisingly, changing a loop’s closing pairs can affect small molecule affinity [44, 45]. Many motifs appeared only once, providing a potential specific target site for small molecules. Further analysis was only completed on bulges and internal loops since the diversity of the hairpin loops was too large (see bar labeled “others” in Fig. 3a) and the sample size of multibranch loops is too small (17 motifs) for meaningful analysis (Fig. 3a).

### General survey of motifs in other types of RNAs

The motifs present in other RNAs are also diverse. There are a total of 26213 non-canonically paired motifs: 6937 bulges, 8457 internal loops, and 10819 hairpins. For highly expressed human RNAs with known structures, there are 2712 total motifs including 157 5’ bulges, 123 3’ bulges, 378 internal loops, 1521 hairpins, and 534 multibranch loops. Differences were observed in the distribution of motifs between other types of RNAs and human miRNAs. For example, the percentage of large hairpins is significantly less in other RNAs as compared to miRNAs (Fig. 3b). In contrast, the percentage of 4-nucleotide hairpins and 2-nucleotide bulges in much greater (Fig. 3b).

### Small loops prevail in bulges and internal loops

As listed in Table 1 and shown in Fig. 3, the most highly represented bulges and internal loops for precursor miRNAs are the smallest possible size: 1-nucleotide bulges and 1 × 1 nucleotide internal loops. Specifically, 69.3 % of 5’ bulge loops and 71.4 % of 3’ bulge loops are one-nucleotide bulges. Not surprisingly, the four possible 1-nucleotide bulges are the four most prevalent bulge loops. Two-nucleotide bulges are next most prevalent (15.0 % for 5’ bulge and 12.6 % for 3’ bulge). Likewise, small bulges and internal loops prevail in other types of RNAs and highly expressed human RNAs. For example, 1- and 2-nucleotide bulges account for ~92 % of all bulges of other RNAs and 85 % of human RNAs.

**Table 1:** The 20 most frequent 5’ bulges, 3’ bulges, and internal loop

	5’ Bulge			3’ Bulge			Internal Loops		
#	Unpaired	Count	Fraction^a^	Unpaired	Count	Fraction^a^	Unpaired	Count	Fraction^a^
1	U	257	0.236	A	215	0.233	G/G	325	0.084
2	A	212	0.195	U	195	0.211	A/C	295	0.076
3	C	163	0.150	C	165	0.179	C/A	290	0.075
4	G	123	0.113	G	85	0.092	U/U	288	0.075
5	CU	20	0.018	GA	15	0.016	U/C	220	0.057
6	UU	18	0.017	UU	12	0.013	C/U	199	0.052
7	UC	16	0.015	AU	11	0.012	A/A	147	0.038
8	UA	13	0.012	UC	9	0.010	C/C	130	0.034
9	GU	12	0.011	AA	8	0.009	G/A	130	0.034
10	AA	11	0.010	CC	8	0.009	A/G	113	0.029
11	CA	11	0.010	CA	7	0.008	UU/UU	26	0.007
12	CC	11	0.010	GU	7	0.008	CA/CA	21	0.005
13	GA	11	0.010	UA	7	0.008	GUUG/AA	15	0.004
14	AU	10	0.009	CU	6	0.006	A/GG	14	0.004
15	AC	9	0.008	AC	5	0.005	UU/U	14	0.004
16	AG	7	0.006	GC	5	0.005	U/CU	13	0.003
17	UG	7	0.006	GG	5	0.005	GA/A	12	0.003
18	GG	4	0.004	UG	5	0.005	AG/G	11	0.003
19	UCAACA	4	0.004	AAA	4	0.004	UU/CU	11	0.003
20	ACC	3	0.003	CUU	4	0.004	A/GC	10	0.003

^a^fraction of the unpaired nucleotide sequence (loop) in its category (5’ bulge, 3’ bulge, or internal loop)

For internal loops in precursor miRNAs, 55.4 % of the 3860 internal loops are 1 × 1 nucleotide internal loops. The second most prevalent internal loop size is 2 × 2 (11.2 %) followed by 1 × 2 and 2 × 1 internal loops (8.9 %) (Fig. 3a). This overall trend is similar for other RNAs: 1 × 1 loops account for 39.8 % of all loops while 2 × 2 and 1 × 2 / 2 × 1 nucleotide loops account for 11.8 % and 15.1 %, respectively. In highly expressed human RNAs, 1 × 1 loops account for 49.7 % of all loops while 2 × 2 and 1 × 2 / 2 × 1 nucleotide loops account for 6.9 % and 7.7 %, respectively. Since smaller bulges and internal loops are thermodynamically more stable than their larger counterparts [46–51], it is not surprising that they are more highly represented.

### Nucleotide preferences in single nucleotide 5’ bulge and 3’ bulge loops in precursor miRNAs

From thermodynamic studies, 1-nucleotide pyrimidine bulges (C or U) are more stable than 1-nucleotide purine bulges (A or G) independent of bulge position (5’ or 3’) [51]. Thus, one might expect that pyrimidine bulges would occur more frequently than purine bulges and that the position of the bulge (5’ or 3’) would not influence the order of frequency. In order to investigate if miRNA hairpin precursors have a preference for certain nucleotides and if this preference is position-dependent, we employed a pooled population comparison, a statistical approach that affords a confidence interval that the preference is not random (see Methods). For example, when “Motif 1” occurs with a certain probability within a given sample size, a random distribution assumes that “Motif 2” occurs with a similar probability. To reject this hypothesis, a Z-score is calculated, which represents the confidence that an increased or decreased frequency of a motif did not occur randomly and thus is truly enriched or depleted.

**Fig. 4:**
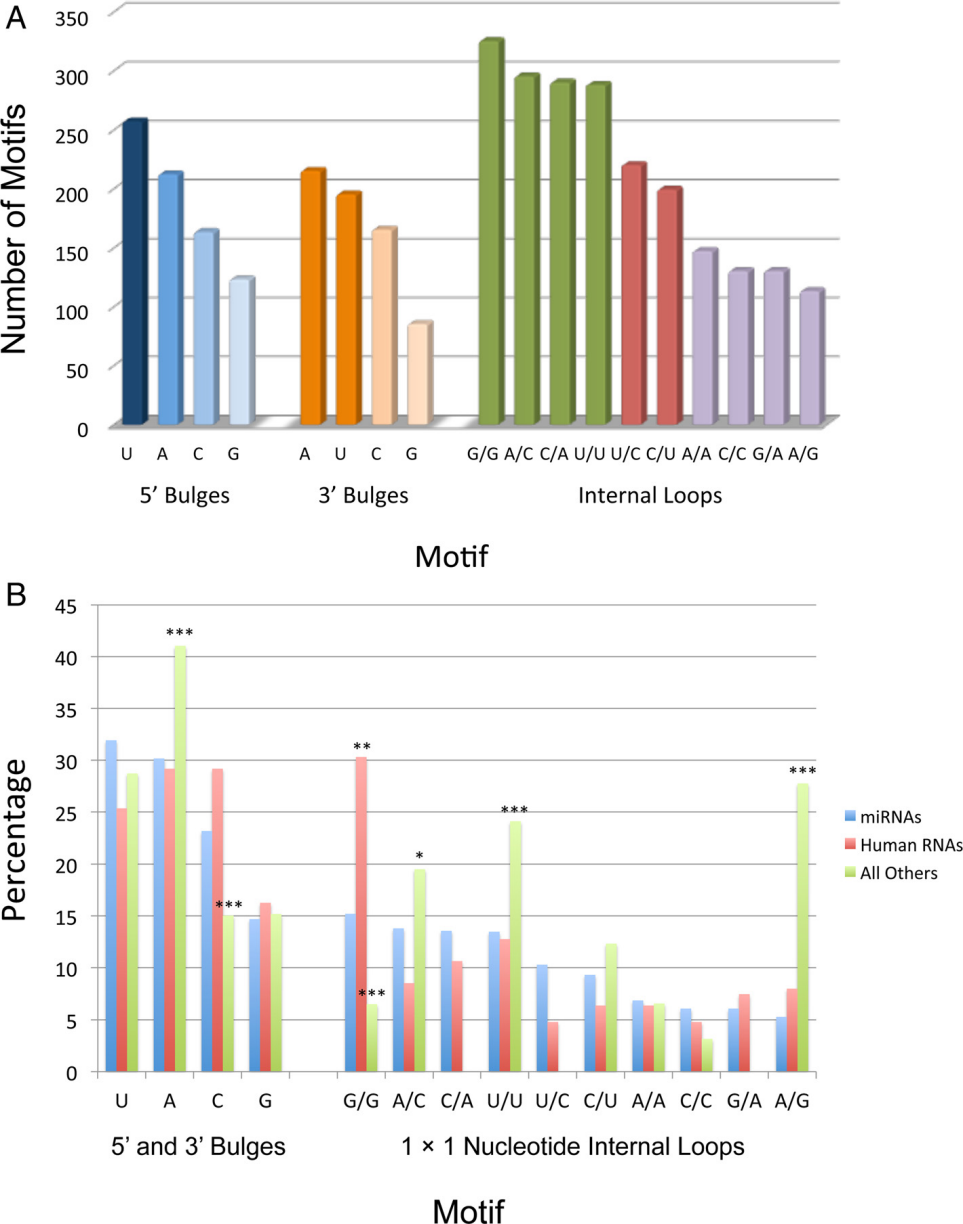
Plot of the number of the most frequently occurring 5’ bulges, 3’ bulges, and internal loops without considering closing base pairs in miRNA precursors and all other RNAs. (**a**) distribution in miRNAs. In each group, different colors indicate that the difference in the rate of occurrence is statistically significant. (**b**) comparison of miRNAs to highly expressed human RNAs and RNAs from other organisms. *, *p* <0.05; **, *p* <0.01; ***, *p* < 0.001

As shown in Fig. 4 and listed in Table 1, the order of single nucleotide occurrence in 5’ bulges is U > A > C > G while in 3’ bulges the order is A ≈ U > C > G (Table 1 and Fig. 4). (Please note that “>” indicates the two frequencies of occurrence are significantly different with Z-score >2 while “≈” indicates Z-score <2). These orders are not correlated to the order of 1-nucleotide bulge thermodynamic stabilities (C ≈ U > A ≈ G). Furthermore, the occurrences of U in 5’ bulges and 3’ bulges are similar (0.236 and 0.233, respectively) as is the occurrences of C or G in 5’ bulges and 3’ bulges. However, A occurs more frequently as a 3’ bulge than a 5’ bulge with Z-score = 2.08. For highly expressed human RNAs, the trends are: 5’ bulge nucleotide: A ≈ C ≈ U > G; 3’ bulge nucleotide: A ≈ C > U ≈ G, although none of these differences is statistically significant.

The distribution of nucleotides in 1-nucleotide bulges is similar for human miRNAs and other highly expressed human RNAs; indeed, there are no statistically significant differences between them. In contrast, 1-nucleotide A bulges appear more often in RNAs from other organisms while 1-nucleotide C bulges appear less often (Fig. 4b).

The structure of an RNA in general and bulges in particular [52] can be dynamic, resulting in multiple folds. Thus, the thermodynamically optimal state of an unbound RNA target may not be the same as the three dimensional structure of a protein- or small molecule-bound state. This may be advantageous for targeting RNA as the RNA's structure may remodel to accommodate ligand binding in a conformational selection mechanism.

### Bulges prefer different closing base pairs

**Fig. 5:**
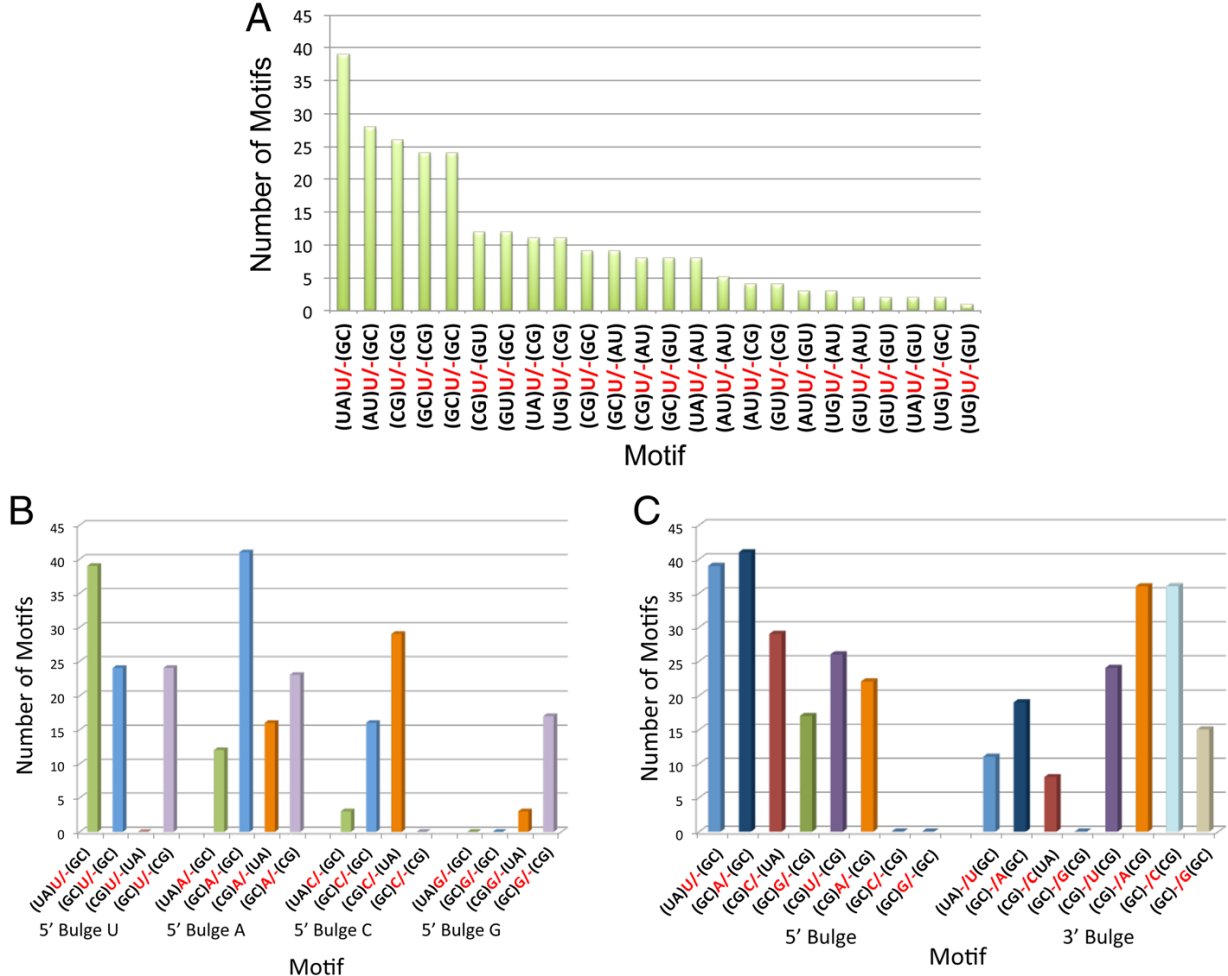
Analysis of bulges found in human miRNAs. (**a**) the occurrences of 5’ bulge U’s with different closing base pair combinations. (**b**) the number of 1-nucleotide 5’ bulges with different closing base pair combinations found in human miRNA hairpin precursors. The most frequently occurring closing base pair combination was determined for each 1-nucleotide bulge, and then calculated for all others. Each closing base pair combination has a unique color, which is applied to each type of bulge. (**c**) the directionality of the motif (5’ bulge vs. 3’ bulge) influences preference of closing base pairs.

For each frequently occurring bulge, there are diverse combinations of closing base pairs, and their frequencies are dependent upon the bulged nucleotide. For example, there are 25 different closing base pair combinations for 5’ bulge U, and the occurrences of these closing pair combinations are different, ranging from 1 to 39 (Fig. 5a).

We analyzed all 5’ 1-nucleotide bulges to determine if there is a preference for the most frequently occurring closing base pair combinations. Figure 5b shows that each 5’ bulge prefers different closing base pair combinations. In some cases, the position of the bulge also influences the preferred closing base pairs; that is, whether it is a 5’ or 3’ bulge (Fig. 5c). For example, 5’ bulge (UA)U/-(GC) occurs 39 times (2^nd^ most prevalent) while 3’ bulge (UA)-/U(GC) occurs only 11 times (7^th^-most prevalent).

As shown schematically in Fig. 2, the same motif (including closing base pairs) could be placed in different orientations in the miRNA's structure. Since their thermodynamic stabilities are the same, we inquired if the direction affects the frequency of occurrence. For example, 5’ bulge (UA)U/-(GC) is the same as 3’ bulge (CG)-/U(AU). The 5’ bulge (UA)U/-(GC) was observed 39 times in human miRNAs (the most frequent base pair combination). However, the 3’ bulge (CG)-/U(AU) was not observed.

There are examples in which the directionality of a motif does not affect occurrence. For example, 5’ bulge (GC)U/-(GC) occurs 24 times; the corresponding 3’ bulge, (CG)-/U(CG), also occurs 24 times. A more sophisticated analysis will be required in order to determine why directionality matters for some motifs but not others.

As observed for miRNA precursors, each 5’ and 3’ 1-nucleotide bulge in highly expressed human RNAs has a different distribution of observed closing base pairs (Additional file 1: Figure S1). Because of the small sample size (*n* = 88 for 3’ bulges and *n* = 121 for 5’ bulges), statistically significant differences were not observed. The most frequently occurring 5’ bulges were (UA)A/-(GC) (*n* = 11) while the most frequently occurring 3’ bulge was (UG)-/G(UA) (*n* = 7). Interestingly, the 5’ bulge (UA)A/-(GC) was not observed as a 3’ bulge (CG)-/A(AU). Another frequently occurring 5’ bulge, (GC)U/- (GC) (*n* = 7) was also not observed as a 3’ bulge. The most frequently occurring 3’ bulge, (UG)-/G(UA), was only observed once as the corresponding 5’ bulge.

### Nucleotide preferences for 1 × 1 nucleotide internal loops

The ten possible 1 × 1 nucleotide internal loops are the ten most frequently occurring internal loops in miRNA hairpin precursors (Table 1). They can be divided into three groups based on their frequencies of occurrence. In order for an internal loop to be placed in a particular group, its Z-score > 2 when compared to the loops in the other groups (Table 2 and Fig. 4). Group 1 contains the most frequently occurring loops including G/G, A/C, C/A, and U/U; Group 2 (second most frequently occurring) includes U/C and C/U; and Group 3 (least frequently occurring) includes A/A, C/C, G/A, and A/G. It is important to point out A/C and C/A are the same motifs but different orientations as are U/C and C/U, and G/A and A/G. Evidently, the direction of the unpaired nucleotides does not matter. For 1 × 1 nucleotide loops in which both nucleotides are the same, the order of occurrence is G/G ≈ U/U > A/A ≈ C/C, which is different from the order observed for bulge loops.

**Table 2:** Relative Z-scores for the occurrences of different 1 × 1 nucleotide internal loops (no consideration of closing base pairs)

Z‐score	G/G	A/C	C/A	U/U	U/C	C/U	A/A	C/C	G/A	A/G
G/G	0.00	1.26	1.47	1.56	4.67	5.70	8.46	9.42	9.42	10.43
A/C	−1.26	0.00	0.22	0.30	3.42	4.46	7.25	8.23	8.23	9.26
C/A	−1.47	−0.22	0.00	0.09	3.21	4.25	7.04	8.03	8.03	9.06
U/U	1.56	−0.30	−0.09	0.00	3.12	4.17	6.96	7.95	7.95	8.98
U/C	−4.67	−3.42	−3.21	−3.12	0.00	1.05	3.90	4.92	4.92	5.99
C/U	5.70	−4.46	−4.25	−4.17	−1.05	0.00	2.86	3.89	3.89	4.97
A/A	−8.46	−7.25	−7.04	−6.96	−3.90	−2.86	0.00	1.04	1.04	2.15
C/C	−9.42	−8.23	−8.03	−7.95	−4.92	−3.89	−1.04	0.00	0.00	1.11
G/A	−9.42	−8.23	−8.03	−7.95	−4.92	−3.89	−1.04	0.00	0.00	1.11
A/G	−10.43	−9.26	−9.06	−8.98	−5.99	−4.97	−2.15	−1.11	−1.11	0.00

Differences in frequency are observed when comparing 1 × 1 nucleotide internal loops in highly expressed human RNAs and other RNAs. For example, G/G loops appear more frequently in highly expressed human RNAs and less frequently in RNAs from other organisms as compared to miRNAs. A/C, A/G, and U/U loops appear more frequently in other RNAs than in miRNA precursors.

### 1 × 1 nucleotide internal loops also have preferences for closing base pairs

**Fig. 6:**
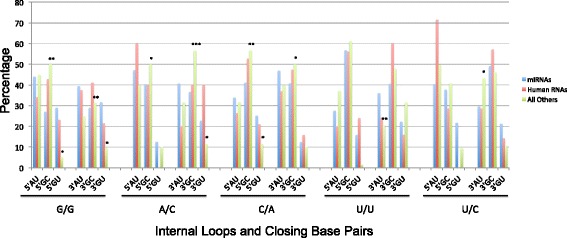
The five most frequently occurring 1×1 nucleotide internal loops in human miRNA precursors have different preferences for 5’ and 3’ closing base pairs. Please note that “5’AU” indicates a 5’AU or 5’UA closing base pair. Likewise, “5’GC” indicates a 5’GC or 5’CG closing base pair, and “5’GU” indicates a 5’GU or 5’UG closing base pair. Interestingly, changing the orientation of the loop nucleotides changes closing base pair preferences. For example, in miRNAs, C/A prefers 5’ GC > AU > GU and 3’ AU > GC > GU while A/C prefers 5’ AU > GC > GU and 3’ AU ≈ GC > GU. The distribution of closing pairs is different for highly expressed human RNAs and RNAs from other organisms. Statistically significant differences were observed for RNAs from other organisms as indicated by *, *p* <0.05; **, *p* <0.01; ***, *p* < 0.001

Previous studies have shown that loop closing base pairs affect loop thermodynamic stability and structure [46, 48, 49]. We therefore investigated if the five most frequently occurring 1 × 1 nucleotide loops (G/G, A/C, C/A, U/U, and U/C) in miRNAs have closing base pair preferences. In this analysis, AU and UA, GC and CG, and GU and UG closing base pairs were grouped together. (Thus, AU indicates AU and UA closing pairs; GC indicates GC and CG closing pairs; and GU indicates GU and UG closing pairs.) The results are summarized in Fig. 6. Interestingly, G/G, A/C, and U/C have the same order of preference for 5’ closing base pairs: AU > GC > GU. C/A and U/U prefer GC > AU > GU for the 5’ closing pair. In contrast, A/C, U/U, and U/C have the same 3’ closing base pair preferences: GC > AU > GU. Unique trends are observed for G/G (AU > GC ≈ GU) and C/A (AU ≈ GC > GU).

As was observed with bulges, directionality affects frequency in some cases. For example, C/A and A/C internal loops have different preferences for the 5’ closing base pair. Similarly, internal loop (UA)C/A(GC) and (CG)A/C(AU) are the same loop. However, (UA)C/A(GC) occurs 29 times while internal loop (CG)A/C(AU) occurs 14 times. The difference in the frequency of occurrence is statistically significant (Z-score = 2.32).

Since the most frequently occurring 1 × 1 nucleotide loops were similar in highly expressed human RNAs and RNAs from other organisms with known structures, we also studied closing base pair preferences for those RNAs. Unlike miRNA precursors, the five loops each have unique preferences for 5’ and 3’ closing base pairs (Fig. 6). For highly expressed human RNAs, an analysis of the closing base pairs of all 1 × 1 nucleotide loops reveals that GU closing pairs are discriminated against as both 5’ and 3’ closing pairs as compared to GC pairs (Z-score = 2.92 and 2.77, respectively). There is no statistically significant difference between GC and AU closing pairs or between AU and GU closing pairs. There are statistically significant differences in the closing base pairs for the five loops when comparing human miRNA precursors to RNAs from other organisms (*n* = 12; Fig. 6). The most statistically significant difference is the preference for 3’GC closing pairs for A/C internal loops (*p* < 0.0001).

### MiRNA processing sites

Presumably, the functionally important sites in miRNA hairpin precursors are the processing sites, where precursors are cleaved by Dicer and Drosha to form the mature miRNA. How do Dicer and Drosha determine the exact sites to cleave? Are they chosen by a specific sequence, motif, or proximity to up/downstream elements? We therefore analyzed the secondary structures of Dicer and Drosha processing sites.

The site corresponding to the 5’ end of the mature RNA is referred to as the start processing site while the 3’ end of the mature RNA is referred to as the end processing site (Fig. 1). The processing site nucleotide can be paired (including loop closing base pairs), a bulged nucleotide, an internal loop nucleotide, a hairpin nucleotide, or at the terminal ends. Of all start processing site nucleotides, 57.7 % are paired (including loop closing pairs) while 49.0 % of end processing site nucleotides are paired. This difference is statistically significant; that is, it can be stated that start processing site nucleotides occur more frequently as paired than end processing site nucleotides do (Z-score = 4.68). There are also a small number of processing sites in terminal ends—17 start processing sites and 28 end processing sites.

We next determined the number of unique motifs that reside in Dicer and Drosha processing sites. If considering only loop nucleotides, there are 507 unique Dicer (*n* = 334) and Drosha (*n* = 173) processing sites. This corresponds to 17.8 % of all processing sites, 21.7 % of all unique miRNA motifs, and 6.8 % of all miRNA motifs. Of the 507 unique Dicer and Drosha processing sites, 39 are present in highly expressed human RNAs. If closing base pairs also confer uniqueness, then there are 752 unique Dicer (*n* = 451) and Drosha (*n* = 301) sites, corresponding to 26.4 % of all processing sites, 19.7 % of all unique miRNA motifs, and 10.1 % of all miRNA motifs. The majority of unique Dicer processing sites reside in internal loops (38.4 % when considering closing base pairs) or hairpins (44.3 % when considering closing base pairs), while the majority of unique Drosha sites reside in internal loops (85.4 % when considering closing base pairs). Of these sites, 742 are unique to human miRNAs as compared to highly expressed human RNAs.

## Conclusions

In this study, we constructed a database of the secondary structural elements (motifs) found in human miRNA hairpin precursor secondary structures. Analysis of this database reveals that small loops prevail in bulges and internal loops. Interestingly, loops and bulges have significantly different preference for loop nucleotides, which also dictate preference for closing base pairs and closing base pair combinations. The origins of these preferences are not clear, but they likely affect the binding of proteins and small molecules. We also examined the motifs present at miRNA processing sites. More than half of the 5’ (start) and 3’ (end) processing sites are in paired regions. Hopefully, the database and its analysis will facilitate the development of small molecules that specifically bind and modulate miRNA function, in particular, those that are associated with cancer or other diseases.

## Additional file

Additional file 1: Figure S1. Analysis of the closing base pairs for 1-nucleotide bulges, both 5’ and 3’, in highly expressed human RNAs with known structures. As observed for 5’ and 3’ bulges in miRNA precursors, each bulge has preferred 5’ and 3’ closing base pairs. Further, the distribution of closing base pairs is different for miRNA precursors and other human RNAs (Fig. 5). (PDF 319 kb)

## References

[1] Krol J, Loedige I, Filipowicz W (2010). The widespread regulation of microRNA biogenesis, function and decay. Nat Rev Genet.

[2] Kim VN, Han J, Siomi MC (2009). Biogenesis of small RNAs in animals. Nat Rev Mol Cell Bio.

[3] Jones-Rhoades MW, Bartel DP, Bartel B (2006). MicroRNAs and their regulatory roles in plants. Annu Rev Plant Biol.

[4] He L, Hannon GJ. MicroRNAs: Small RNAs with a big role in gene regulation. Nat Rev Genet. 2004;5(7):522–31. 10.1038/nrg1379 15211354

[5] Cui QH, Lu M, Zhang QP, Deng M, Miao J, Guo YH, et al. An analysis of human microRNA and disease associations. PLoS One. 2008;3(10):e3420. 10.1371/journal.pone.0003420 PMC2559869 18923704

[6] Sander C, Betel D, Wilson M, Gabow A, Marks DS (2008). The microRNA.org resource: targets and expression. Nucleic Acids Res.

[7] Calin GA, Croce CM (2006). MicroRNA signatures in human cancers. Nat Rev Cancer.

[8] Olson EN, Small EM (2011). Pervasive roles of microRNAs in cardiovascular biology. Nature.

[9] Benkirane M, Triboulet R, Mari B, Lin YL, Chable-Bessia C, Bennasser Y, et al. Suppression of microRNA-silencing pathway by HIV-1 during virus replication. Science. 2007;315(5818):1579–82. 10.1126/science.1136319 17322031

[10] Huang J, Wang F, Argyris E, Chen K, Liang Z, Tian H, et al. Cellular microRNAs contribute to HIV-1 latency in resting primary CD4+ T lymphocytes. Nat Med. 2007;13(10):1241–7. 10.1038/nm1639 17906637

[11] John B, Enright AJ, Aravin A, Tuschl T, Sander C, Marks DS. Human microRNA targets (vol 2, pg 1862, 2005). PLoS Biol. 2005;3(7):1328–8. 10.1371/journal.pbio.0020363 PMC521178 15502875

[12] Garzon R, Marcucci G, Croce CM (2010). Targeting microRNAs in cancer: rationale, strategies and challenges. Nat Rev Drug Discov.

[13] Calin GA, Zhang S, Chen L, Jung EJ. Targeting microRNAs with small molecules: from dream to reality. Clin Pharmacol Ther. 2010;87(6):754–8. 10.1038/clpt.2010.46 PMC3902962 20428111

[14] Petrocca F, Visone R, Onelli MR, Shah MH, Nicoloso MS, de Martino I, et al. E2F1-regulated microRNAs impair TGFbeta-dependent cell-cycle arrest and apoptosis in gastric cancer. Cancer Cell. 2008;13(3):272–86. 10.1016/j.ccr.2008.02.013 18328430

[15] Frankel LB, Christoffersen NR, Jacobsen A, Lindow M, Krogh A, Lund AH (2008). Programmed cell death 4 (PDCD4) is an important functional target of the microRNA miR-21 in breast cancer cells. J Biol Chem.

[16] Meng F, Henson R, Wehbe-Janek H, Ghoshal K, Jacob ST, Patel T (2007). MicroRNA-21 regulates expression of the PTEN tumor suppressor gene in human hepatocellular cancer. Gastroenterology.

[17] Aagaard L, Rossi JJ (2007). RNAi therapeutics: principles, prospects and challenges. Adv Drug Deliv Rev.

[18] Loya CM, Lu CS, Van Vactor D, Fulga TA (2009). Transgenic microRNA inhibition with spatiotemporal specificity in intact organisms. Nat Methods.

[19] Bose D, Jayaraj G, Suryawanshi H, Agarwala P, Pore SK, Banerjee R, et al. The tuberculosis drug streptomycin as a potential cancer therapeutic: inhibition of miR-21 function by directly targeting its precursor. Angew Chem Int Ed Engl. 2012;51(4):1019–23. 10.1002/anie.201106455 22173871

[20] Velagapudi SP, Disney MD (2014). Two-dimensional combinatorial screening enables the bottom-up design of a microRNA-10b inhibitor. Chem Commun (Camb).

[21] Velagapudi SP, Gallo SM, Disney MD (2014). Sequence-based design of bioactive small molecules that target precursor microRNAs. Nat Chem Biol.

[22] Thomas JR, Hergenrother PJ (2008). Targeting RNA with small molecules. Chem Rev.

[23] Guan L, Disney MD (2012). Recent advances in developing small molecules targeting RNA. ACS Chem Biol.

[24] Tran T, Disney MD (2010). Two-dimensional combinatorial screening of a bacterial rRNA A-site-like motif library: defining privileged asymmetric internal loops that bind aminoglycosides. Biochemistry.

[25] Ambros V, Bartel B, Bartel DP, Burge CB, Carrington JC, Chen X, et al. A uniform system for microRNA annotation. RNA. 2003;9(3):277–9. 10.1261/rna.2183803 PMC1370393 12592000

[26] Lee Y, Jeon K, Lee JT, Kim S, Kim VN. MicroRNA maturation: stepwise processing and subcellular localization. EMBO J. 2002; 21(17):4663-70. 10.1093/emboj/cdf476 PMC126204 12198168

[27] Griffiths-Jones S, Kozomara A (2011). miRBase: integrating microRNA annotation and deep-sequencing data. Nucleic Acids Res.

[28] Griffiths-Jones S, Saini HK, van Dongen S, Enright AJ (2008). miRBase: tools for microRNA genomics. Nucleic Acids Res.

[29] Mathews DH, Disney MD, Childs JL, Schroeder SJ, Zuker M, Turner DH (2004). Incorporating chemical modification constraints into a dynamic programming algorithm for prediction of RNA secondary structure. Proc Natl Acad Sci U S A.

[30] Mathews DH, Turner DH (2006). Prediction of RNA secondary structure by free energy minimization. Curr Opin Struct Biol.

[31] Mathews DH, Sabina J, Zuker M, Turner DH (1999). Expanded sequence dependence of thermodynamic parameters improves prediction of RNA secondary structure. J Mol Biol.

[32] Gutell RR (1994). Collection of small subunit (16S- and 16S-like) ribosomal RNA structures: 1994. Nucleic Acids Res.

[33] Schnare MN, Damberger SH, Gray MW, Gutell RR (1996). Comprehensive comparison of structural characteristics in eukaryotic cytoplasmic large subunit (23 S-like) ribosomal RNA. J Mol Biol.

[34] Szymanski M, Barciszewska MZ, Erdmann VA, Barciszewski J. 5S ribosomal RNA database. Nucleic Acids Res. 2002;30(1):176–8. 10.1093/nar/30.1.176 PMC99124 11752286

[35] Sprinzl M, Horn C, Brown M, Ioudovitch A, Steinberg S (1998). Compilation of tRNA sequences and sequences of tRNA genes. Nucleic Acids Res.

[36] Larsen N, Samuelsson T, Zwieb C (1998). The signal recognition particle database (SRPDB). Nucleic Acids Res.

[37] Brown JW (1998). The ribonuclease P database. Nucleic Acids Res.

[38] Damberger SH, Gutell RR (1994). A comparative database of group I intron structures. Nucleic Acids Res.

[39] Waring RB, Davies RW (1984). Assessment of a model for intron RNA secondary structure relevant to RNA self-splicing--a review. Gene.

[40] Michel F, Umesono K, Ozeki H (1989). Comparative and functional anatomy of group II catalytic introns--a review. Gene.

[41] Juhling F, Morl M, Hartmann RK, Sprinzl M, Stadler PF, Putz J (2009). tRNAdb 2009: compilation of tRNA sequences and tRNA genes. Nucleic Acids Res.

[42] SantaLucia Jr J, Turner DH. Structure of (rGGCGAGCC)_2_ in solution from NMR and restrained molecular dynamics. Biochemistry. 1993;32(47):12612–23. 10.1021/bi00210a009 8251479

[43] Wu M, Turner DH. So-lution structure of (rGCGGACGC)_2_ by two-dimensional NMR and the iterative relaxation matrix approach. Biochemistry. 1996;35(30):9677–89. 10.1021/bi960133q 8703939

[44] Pushechnikov A, Lee MM, Childs-Disney JL, Sobczak K, French JM, Thornton CA, et al. Rational design of ligands targeting triplet repeating transcripts that cause RNA dominant disease: application to myotonic muscular dystrophy type 1 and spinocerebellar ataxia type 3. J Am Chem Soc. 2009;131(28):9767–79. 10.1021/ja9020149 PMC2731475 19552411

[45] Tran T, Disney MD (2011). Molecular recognition of 6’-N-5-hexynoate kanamycin A and RNA 1x1 internal loops containing CA mismatches. Biochemistry.

[46] Chen G, Znosko BM, Jiao X, Turner DH (2004). Factors affecting thermodynamic stabilities of RNA 3 x 3 internal loops. Biochemistry.

[47] Freier SM, Kierzek R, Jaeger JA, Sugimoto N, Caruthers MH, Neilson T, et al. Improved free-energy parameters for predictions of RNA duplex stability. Proc Natl Acad Sci U S A. 1986;83(24):9373–7. 10.1073/pnas.83.24.9373 PMC387140 2432595

[48] Schroeder SJ, Burkard ME, Turner DH (1999). The energetics of small internal loops in RNA. Biopolymers.

[49] Schroeder SJ, Turner DH (2001). Thermodynamic stabilities of internal loops with GU closing pairs in RNA. Biochemistry.

[50] Zhu J, Wartell RM (1999). The effect of base sequence on the stability of RNA and DNA single base bulges. Biochemistry.

[51] Znosko BM, Silvestri SB, Volkman H, Boswell B, Serra MJ (2002). Thermodynamic parameters for an expanded nearest-neighbor model for the formation of RNA duplexes with single nucleotide bulges. Biochemistry.

[52] Stelzer AC, Frank AT, Kratz JD, Swanson MD, Gonzalez-Hernandez MJ, Lee J, et al. Discovery of selective bioactive small molecules by targeting an RNA dynamic ensemble. Nat Chem Biol. 2011;7(8):553–9. 10.1038/nchembio.596 PMC3319144 21706033

