# Supplementary material for: Analysis of secondary structural elements in human microRNA hairpin precursors
Source: BMC Bioinformatics. 2016 Mar 1;17:112. doi: 10.1186/s12859-016-0960-6 (PMC4772329; doi:10.1186/s12859-016-0960-6)
Supplement: Additional file 1: Figure S1. — Analysis of the closing base pairs for 1-nucleotide bulges, both 5’ and 3’, in highly expressed human RNAs with known structures. As observed for 5’ and 3’ bulges in miRNA precursors, each bulge has preferred 5’ and 3’ closing base pairs. Further, the distribution of closing base pairs is different for miRNA precursors and other human RNAs (Fig. 5). (PDF 319 kb) [file 12859_2016_960_MOESM1_ESM.pdf]

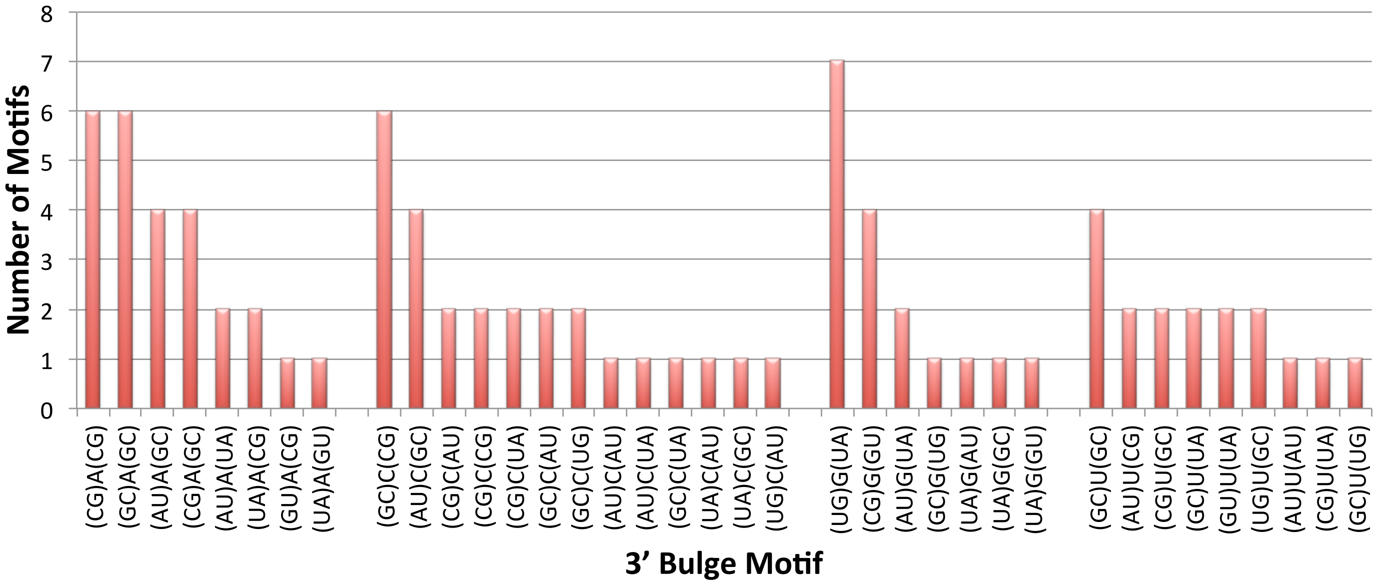
Number of Motifs
8
7
6
5
4
3
2
1
0
(CG)A(CG)
(GC)A(GC)
(AU)A(GC)
(CG)A(GC)
(AU)A(UA)
(UA)A(CG)
(GU)A(CG)
(UA)A(GU)
(GC)C(CG)
(AU)C(GC)
(CG)C(AU)
(CG)C(CG)
(CG)C(UA)
(GC)C(AU)
(GC)C(UG)
(AU)C(AU)
(AU)C(UA)
(GC)C(UA)
(UA)C(AU)
(UA)C(GC)
(UG)C(AU)
(UG)G(UA)
(CG)G(GU)
(AU)G(UA)
(GC)G(UG)
(UA)G(AU)
(UA)G(GC)
(UA)G(GU)
(GC)U(GC)
(AU)U(CG)
(CG)U(GC)
(GC)U(UA)
(GU)U(UA)
(UG)U(GC)
(AU)U(AU)
(CG)U(UA)
(GC)U(UG)
3’ Bulge Motif

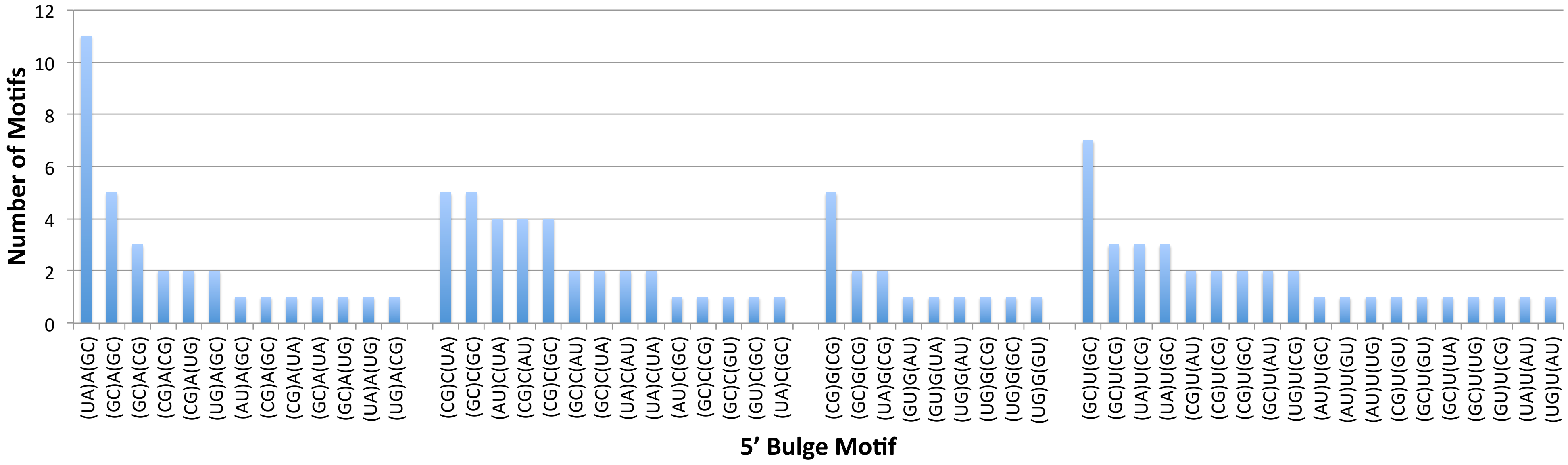
Number of Motifs
12
10
8
6
4
2
0
(UA)A(GC)
(GC)A(GC)
(GC)A(CG)
(CG)A(CG)
(CG)A(UG)
(UG)A(GC)
(AU)A(GC)
(CG)A(GC)
(CG)A(UA)
(GC)A(UA)
(GC)A(UG)
(UA)A(UG)
(UG)A(CG)
(CG)C(UA)
(GC)C(GC)
(AU)C(UA)
(CG)C(AU)
(CG)C(GC)
(GC)C(AU)
(GC)C(UA)
(UA)C(AU)
(UA)C(UA)
(AU)C(GC)
(GC)C(CG)
(GC)C(GU)
(GU)C(GC)
(UA)C(GC)
(CG)G(CG)
(GC)G(CG)
(UA)G(CG)
(GU)G(AU)
(GU)G(UA)
(UG)G(AU)
(UG)G(CG)
(UG)G(GC)
(UG)G(GU)
(GC)U(GC)
(GC)U(CG)
(UA)U(CG)
(UA)U(GC)
(CG)U(AU)
(CG)U(CG)
(CG)U(GC)
(GC)U(AU)
(UG)U(CG)
(AU)U(GC)
(AU)U(GU)
(AU)U(UG)
(CG)U(GU)
(GC)U(GU)
(GC)U(UA)
(GC)U(UG)
(GU)U(CG)
(UA)U(AU)
(UG)U(AU)
5’ Bulge Motif

Figure S1: Analysis of the closing base pairs for 1-nucleotide bulges, both 5’ and 3’, in highly expressed human RNAs with known structures. As observed for 5’ and 3’ bulges in miRNA precursors, each bulge has preferred 5’ and 3’ closing base pairs. Further, the distribution of closing base pairs is different for miRNA precursors and other human RNAs (**Fig. 5**).
